# PRR11 Promotes Bladder Cancer Growth and Metastasis by Facilitating G1/S Progression and Epithelial‐Mesenchymal Transition

**DOI:** 10.1002/cam4.70749

**Published:** 2025-03-10

**Authors:** Lu Wang, Zengshun Kou, Jiaxi Zhu, Xiu Zhu, Lei Gao, Hai Zhu

**Affiliations:** ^1^ Department of Urology, Qingdao Municipal Hospital University of Health and Rehabilitation Sciences Qingdao China; ^2^ Qingdao Municipal Hospital Qingdao University Qingdao China; ^3^ Department of Urology Zhongnan Hospital of Wuhan University Wuhan China; ^4^ Life Sciences, Faculty of Arts & Science University of Toronto‐St. George Campus Toronto Canada; ^5^ Faculty of Information Science and Engineering Ocean University of China Qingdao China

**Keywords:** biomarkers, bladder cancer, cell cycle, PRR11, tumor treatment

## Abstract

**Background:**

Although Proline‐rich Protein 11 (PRR11) abnormalities are closely associated with carcinogenesis, the precise mechanism of bladder cancer remains unclear. Here, we sought to elucidate the molecular mechanisms of PRR11 in bladder cancer.

**Methods:**

We performed differential expression analysis of PRR11 from the TCGA and GEO databases, followed by validation with clinical samples. Survival analysis was employed to assess the correlation between PRR11 and patient prognosis. The effects of PRR11 on bladder cancer cells were examined through both in vitro and in vivo experiments. Additionally, Gene Set Enrichment Analysis (GSEA) was used to predict the downstream pathways associated with PRR11, which were further validated through subsequent experiments.

**Results:**

PRR11 is upregulated in bladder cancer and could lead to poor prognosis. In vitro, PRR11 promoted tumor cell proliferation; in vivo, it promoted subcutaneous tumor growth. PRR11 knockdown inhibited its oncogenic function. On the molecular level, PRR11 promotes tumor metastasis by inducing Epithelial‐mesenchymal Transition (EMT). GSEA suggests that PRR11 is strongly linked to the cell cycle, and silencing of PRR11 can achieve anti‐tumor effects by inhibiting CCNE and blocking the G1/S phase transition.

**Conclusions:**

Our study demonstrates that silencing PRR11 can arrest the malignant progression of bladder cancer by inhibiting EMT and blocking the G1/S transition. Targeting PRR11 may provide new insights for targeting cell cycle therapy.

**Abbreviations**
AUC: area under the curve
BLCA: bladder urothelial carcinoma
EMT: epithelial‐mesenchymal transition
GO: gene ontology
GSEA: gene set enrichment analysis
IF: immunofluorescence
IHC: immunohistochemistry
KEGG: Kyoto Encyclopedia of Genes and Genomes
MIBC: muscle‐invasive bladder cancer
MMR: mismatch repair
NCACC: National Collection of Authenticated Cell Cultures
NLS: nuclear localization signal
NMIBC: non‐muscle‐invasive bladder cancer
OS: overall survival
PRR11: proline‐rich Protein 11
ROC: receiver operating characteristic
SKA2: spindle and kinetochore‐associated 2
SNPs: single nucleotide polymorphisms
TURBT: transurethral resection of bladder tumor
ZFD: Zinc finger structural domain

## 1 Introduction

Bladder urothelial carcinoma (BLCA) is one of the most common tumors of the urinary system, ranking as the 9th most common cancer worldwide and the 6th most prevalent among men [1]. Despite significant advancements in the treatment of BLCA, and the widespread use of targeted therapies and immunotherapies in recent years, the survival rate for BLCA has shown only modest improvements over the past three decades [2]. Therefore, further research into the underlying mechanisms of BLCA is essential for improving prognosis.

Precise cell cycle control is essential for normal cellular functions and genetic stability. In tumor cells, the regulatory mechanisms often become abnormal, leading to uncontrolled cell proliferation. With the application of CDK4/6 inhibitors, targeting the cell cycle has already become an effective anticancer strategy [3]. Novel targeted drugs, including those aimed at cyclin‐dependent kinases (CDKs) and CCNA2, are being developed to enhance the success rate and selectivity of cancer treatments [4, 5, 6].

Proline‐rich Protein 11 (PRR11) is a protein‐coding gene closely associated with the cell cycle [7, 8]. Located on chromosome 17q22–23, adjacent to the SKA2, PRR11 is precisely regulated by P53 [9]. It could regulate the S phase and induce premature chromatin condensation [10]. PRR11 also promotes cell proliferation by interacting with E2F1 to enhance the expression of PTTG1 [11, 12]. The stable expression of PRR11 has been reported to maintain stem cell self‐renewal via the MARK signaling pathway [13]. Moreover, PRR11 is potentially associated with various cancers, such as lung, kidney, and breast cancer [12, 13, 14, 15, 16]. For instance, PRR11 promotes lung cancer progression by inducing filamentous pseudopod formation through the recruitment of ARP2/3 complexes [14]. In clear cell renal cell carcinoma (ccRCC), PRR11 activated by c‐Myc induces the degradation of the E2F1 protein and reduces its stability, affecting cell cycle progression [12]. PRR11 amplifies the PI3K signaling pathway, promotes estrogen‐independent proliferation, and confers endocrine resistance in ER‐positive breast cancer [16]. Regulating the expression of multiple signaling pathways affects the cell cycle, apoptosis, and cell migration, all of which are crucial in the development of tumors. In BLCA, PRR11 expression is elevated and correlates with a poor prognosis [17, 18]. Although the oncogenic role of PRR11 in BLCA has been preliminarily revealed, its functions and mechanisms remain largely unclear, warranting further investigation.

Targeting PRR11 may become an effective therapeutic strategy for bladder cancer with critical clinical applications. Therefore, this study aims to provide new options for treating BLCA by thoroughly investigating the mechanism of PRR11.

## 2 Materials and Methods

### 2.1 Data Sources

We extracted the expression data from the TCGA (https://portal.gdc.cancer.gov/) and GTEx (https://www.gtexportal.org/), which were unified by the Toil process [19]. The data for the GSE13507 chip were obtained from GEO (https://www.ncbi.nlm.nih.gov/geo/) [20]. All data matrices were processed using R‐Project (version: 3.40.2). Survival analysis was performed using two statistical methods: Kaplan–Meier (KM) analysis and Cox regression analysis. Relevant patient information is provided in Table S1 and Data S3.

### 2.2 Landscape of Genetic Mutations

To investigate the genetic alterations of PRR11, we accessed the cBioPortal (https://www.cbioportal.org/), which provides a comprehensive resource for analyzing genetic instability across various cancer types [21]. The pan‐cancer mutation profile of PRR11 was analyzed visually with the “maftools” package. The TCGA‐BLCA sequencing data was analyzed by using the Sangerbox (http://sangerbox.com/). The effect of P53 mutations on PRR11 expression was explored using UALCAN (https://ualcan.path.uab.edu/) [22, 23].

### 2.3 Correlation Analysis and Enrichment Analysis

The STRING database (https://cn.string‐db.org/) was utilized to map protein–protein interaction networks (PPIs). We extracted 100 genes related to PRR11 from the GEPIA (http://gepia.cancer‐pku.cn/). Gene ontology (GO) and Kyoto Encyclopedia of Genes and Genomes (KEGG) analyses of the 100 genes were performed in R‐Project. CancerSEA (http://biocc.hrbmu.edu.cn/CancerSEA/) handled single‐cell analysis and performed functional analysis, redefining a total of 14 functional states [24]. Pearson correlation analysis was employed to calculate the statistical correlation of genes with each gene set.

### 2.4 Patients and Clinical Specimens

Bladder cancer tissues were obtained from surgical patients at Zhongnan Hospital, and all patients had not received any anticancer treatment prior to surgery. The research was carried out with permission from the Ethics Committee of Wuhan University. Clinical information on the patients is provided in Table S2.

### 2.5 Immunofluorescence (IF) and Immunohistochemistry (IHC)

Confocal Petri dishes were used to inoculate the cells. Cells were washed three times with ice‐ cold phosphate buffered saline and then fixed with 4% paraformaldehyde. Cells were coated with 1% bovine serum albumin and then sequentially incubated with the indicated antibody and fluorescently labeled secondary antibody (avoiding light for 1 h). Finally, cells were incubated with 0.1 mg/mL DAPI for nuclear staining (avoiding light for 10 min).

All tissues were fixed (10% formalin‐fixed for 24 h), embedded (paraffin‐embedded), sectioned (2–3 μm), and finally attached to slides. After deparaffinization, hydration, and antibody repair, we detected the expression of PRR11. The results were observed using a fluorescence microscope.

### 2.6 Cell Cultures and Transfections

All human‐derived bladder cancer cell lines were obtained from the National Collection of Authenticated Cell Cultures (NCACC). Cultures were maintained in accordance with NCACC's recommended culture medium. Small interfering RNA targeting PRR11 (siPRR11) was transfected into T24 and UC‐UM‐3 cell lines using Lipofectamine 2000 reagent (Invitrogen, USA) and incubated under humid conditions at 37°C and 5% CO_2_ for 48 h. The sh‐PRR11 lentiviral vector was used to infect the UM‐UC‐3 cell line. Appropriate MOI values were selected for viral infection, and cell resistance screening was performed using puromycin to obtain stably expressing cell lines.

### 2.7 Cell Proliferation Assays, Colony Formation Assays, and Transwell Migration Assays

The cells were digested, and the suspension was mixed and inoculated into a 96‐well plate, with 200 μL of cell suspension added to each well (4000 cells per well for UM‐UC‐3 and 3000 cells per well for T24). At 24, 48, 72, 96, and 120 h of incubation, a 96‐well plate was taken out, and 20 μL of MTT solution at 5 mg/mL was added to each well of each treatment group. The cells were incubated in the incubator for 4 h, and their proliferation ability was measured with the MTT assay (Roche, Germany).

For colony formation assays, transfected cells were inoculated in six‐well plates, and 2 mL of culture medium was added to each well. After resting for 9–14 days, the plate was observed under a microscope to assess cell colony formation. A colony was considered well‐formed if it contained more than 50 cells. The plates were washed once with PBS and fixed with 4% paraformaldehyde for 1 h. The plates were then stained with a 0.1% crystal violet solution for over 1 h.

For Transwell migration assays, cells were inoculated in the center of the upper layer of the Transwell chamber. The lower chamber contained serum medium, and a polycarbonate membrane separated the upper and lower layers of culture. The invaded cells were stained with a 10% Giemsa solution, then observed and captured with a light microscope.

### 2.8 Real‐Time PCR, Western Blotting, and Cell Cycle Assay

We isolated and extracted RNA from cells and measured the concentration and purity of each sample using a NanoPhotometer (Implen, Germany). cDNA was synthesized using the ReverTra Ace qRT‐PCR kit (Toyobo, China). A real‐time polymerase chain reaction was performed using iQTMSYBR Green Supermix (Bio‐Rad, China). The complete sequences of the primers used are shown in Table S3.

Cells were fully lysed with a lysate buffer, and total protein was collected. A 10% SDS‐PAGE gel was prepared, and 10 μg of protein was loaded per well. After electrophoresis, the protein was transferred onto a PVDF membrane, which was blocked for 2 h. Incubation was carried out with primary and secondary antibodies, followed by visualization using a chemiluminescent imaging system. The antibodies used in this study are listed in Table S4.

Cells were digested, washed twice with PBS, and incubated in a working solution containing propidium iodide (PI) and a membrane breaker. The cells were resuspended, mixed thoroughly, and incubated at room temperature for 30 min to ensure sufficient DNA staining. Flow cytometry was used to analyze the proportion of cells in each phase of the cell cycle, comparing the differences in cycle distribution among groups.

### 2.9 Animal Experiments

Balb/c‐nude mice (4–5 weeks of age, weighing 18–20 g) were selected and raised at 25°C under sterile conditions. UM‐UC‐3 cells stably transfected with sh‐PRR11 were injected into the right inguinal region of the mice. Four weeks later, the mice were euthanized, and the tumor volume was calculated. Tumors were fixed with paraformaldehyde for immunohistochemical staining, and the expression levels of Ki67 and PRR11 were detected in the tumor tissues of the two groups.

### 2.10 Statistical Analyses

The statistical graphs of the experimental data were produced and plotted in GraphPad 6.0 software. Western blot bands were initially processed by Image Lab software and subsequently processed by Photoshop software. Statistical analysis was performed in SPSS version 22.0 software. All data were expressed as the mean ± standard deviation (mean ± SD). A *t*‐test was used to compare the differences in mean values between two samples, and an analysis of variance was used to compare the differences in mean values among multiple samples. A *p*‐value less than 0.05 is considered significant. *p < 0.05; **p < 0.01; ***p < 0.001; and ****p < 0.0001.

## 3 Results

### 3.1 PRR11 is Overexpressed in BLCA

**FIGURE 1:**
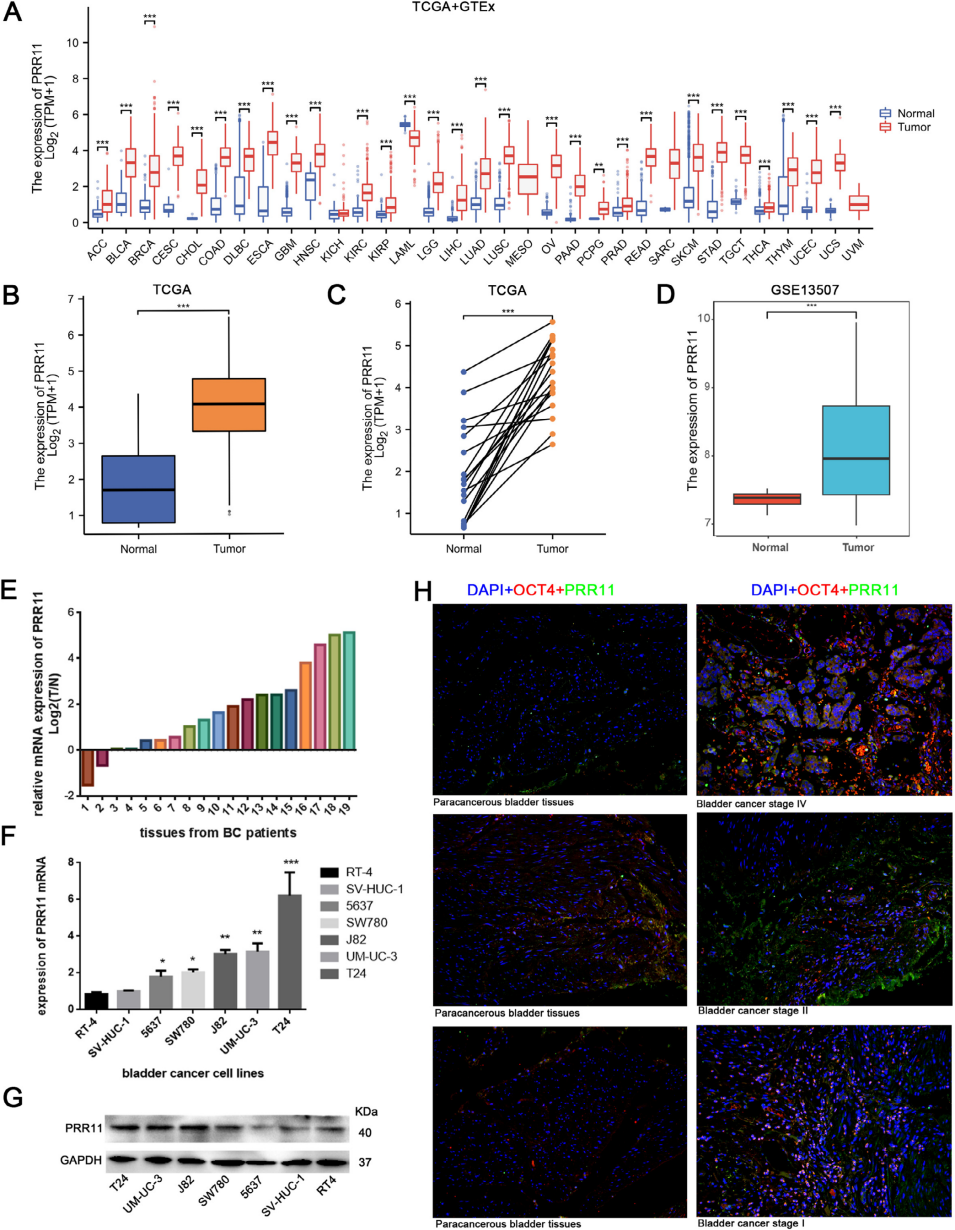
PRR11 promotes the malignant progression of bladder cancer: (A) PRR11 expression in pan‐cancers from TCGA and GTEx databases; PRR11 expression in unpaired (B) and paired samples (C) from the TCGA‐BLCA database; (D) Expression of PRR11 in different samples in the GSE13507 database; (E) RT‐PCR results showed that PRR11 expression was elevated in 17 out of 19 patients; PRR11 mRNA expression (F) and protein expression (G) in seven bladder cell lines; (H) Immunofluorescence staining of tumor tissues and paracancerous tissues at different clinical stages.  *p < 0.05; **p < 0.01; ***p < 0.001; and ****p < 0.0001.

The TCGA and GTEx databases were used for systematic pan‐cancer expression analysis of PRR11. This approach revealed that PRR11 was overexpressed in 28 cancer types, with expression downregulated only in LAML (Figure 1A). In bladder cancer, tumor tissues exhibited higher PRR11 than normal tissues (Figure 1B,C). The GSE13507 dataset verified this result in the GEO database (Figure 1D). In addition, we performed experimental validation in the tumor tissues of patients, and RT‐PCR outcomes showed that PRR11 expression was upregulated in 17 of 19 patients (Figure 1E).

Subsequently, we selected three representative individuals (stages T1, T2, and T4) from the collected tissues of bladder cancer patients and performed immunofluorescence staining of tumor tissues and paracancerous tissues. PRR11 was expressed at elevated levels in tumors, and the trend of elevation was more pronounced in higher stages (Figure 1H). In conclusion, PRR11 is a potential oncogene involved in the development and progression of bladder cancer.

### 3.2 PRR11 Is Associated With a Poor Prognosis

**FIGURE 2:**
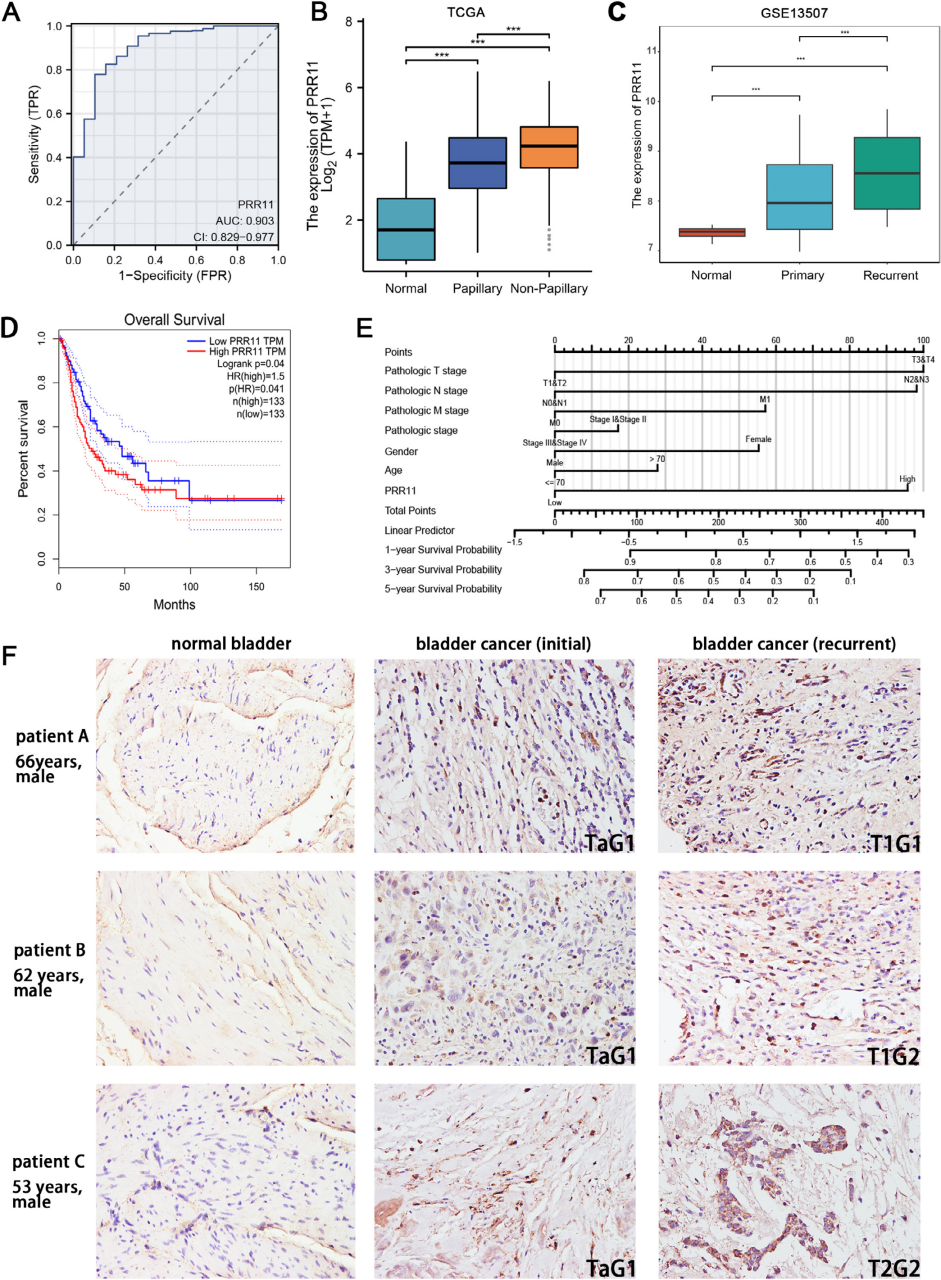
PRR11 overexpression is associated with poor prognosis: (A) ROC curves with AUC showing the diagnostic efficacy of PRR11; (B) Expression of PRR11 in different pathology types from the TCGA‐BLCA database; (C) Differences in PRR11 expression between patients with first diagnosis and recurrent patients from the GSE13507 database; (D) K‐M analysis of OS for PRR11‐LOW and PRR11‐HIGH in BLCA; (E) Nomogram of multifactorial prediction of 1‐, 3‐, and 5‐year OS survival; (F) Immunohistochemical staining from three patients comparing initial TURBT tissue and recurrent tissue (radical cystectomy). *p < 0.05; **p < 0.01; ***p < 0.001; and ****p < 0.0001.

The receiver operating characteristic (ROC) curve demonstrated the utility of PRR11 as a diagnostic marker for BLCA (Figure 2A). Furthermore, PRR11 expression levels were highly correlated with pathological classification, and the expression was higher in non‐papillary tumors (Figure 2B). Next, we analyzed tumor recurrence data in GSE13507 and found that PRR11 expression was significantly elevated in the tumor tissues of recurrent patients compared with those of primary tumors (Figure 2C). Subsequently, we performed validation of the clinical samples, and the PRR11 expression levels of the three patients after recurrence were further elevated compared with those at the initial diagnosis, and the tumor T‐stage and pathological grading also confirmed the progression of the tumors (Figure 2F).

We conducted KM survival analysis to evaluate the clinical prognosis, revealing that PRR11 is a significant prognostic risk factor (Figure 2D and Figure S1A,B). COX regression analyses indicated that PRR11 was an independent risk element (Table 1). To explore the clinical application of PRR11, we constructed a Nomogram for predicting the OS, which included pathological T, N, and M stages and PRR11 as prognostic factors. This Nomogram can predict the probability of OS (Figure 2E and Figure S1C). Above all, PRR11 is correlated with BLCA progression and serves as a predictor of clinical prognosis.

**TABLE 1:** COX regression analysis to assess the impact of PRR11 expression and clinical characteristics on prognosis.

Characteristics	Total (*N*)	Univariate analysis		Multivariate analysis	
		Hazard ratio (95% CI)	*p*	Hazard ratio (95% CI)	*p*
Pathologic T stage	377				
T1&T2	123	Reference		Reference	
T3&T4	254	2.157 (1.485–3.132)	**< 0.001**	2.134 (0.670–6.794)	0.200
Pathologic N stage	367				
N0&N1	284	Reference		Reference	
N2&N3	83	2.228 (1.605–3.093)	**< 0.001**	2.100 (1.185–3.722)	**0.011**
Pathologic M stage	212				
M0	201	Reference		Reference	
M1	11	3.112 (1.491–6.493)	**0.002**	1.355 (0.502–3.661)	0.549
Pathologic stage	409				
Stage I & II	133	Reference		Reference	
Stage III & IV	276	2.267 (1.567–3.281)	**< 0.001**	0.856 (0.257–2.850)	0.800
Gender	411				
Female	108	Reference			
Male	303	0.868 (0.629–1.198)	0.390		
Age	411				
< = 70	231	Reference		Reference	
> 70	180	1.424 (1.064–1.906)	**0.018**	1.244 (0.775–1.996)	0.365
PRR11	411				
Low	205	Reference		Reference	
High	206	1.535 (1.142–2.064)	**0.005**	1.983 (1.224–3.213)	**0.005**

*Note:* The bold numbers indicate statistical significance *p* < 0.05.

### 3.3 Genetic Alterations of PRR11

**FIGURE 3:**
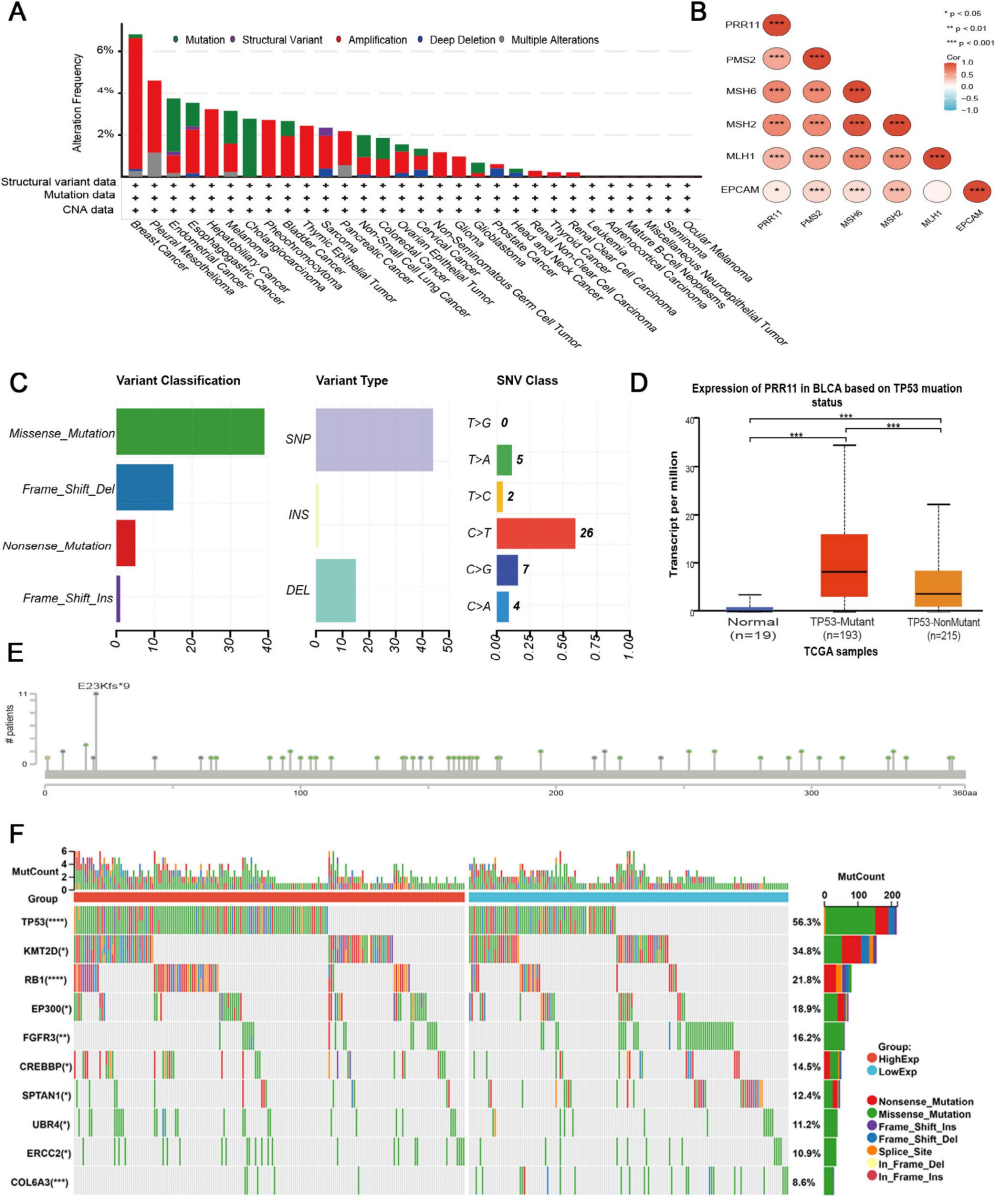
The mutational landscape of PRR11: (A) Pan‐cancer analysis of PRR11 genomic changes from the cBioPortal database, including mutation, amplification, and deep deletion analyses; (B) Heatmap of the correlation between PRR11 and five MMR genes in bladder cancer; (C) Visualization of the pan‐cancer mutation landscape of PRR11 using the maftools package plotmafSummary function in R‐Project; (D) Effect of TP53 mutation on PRR11 expression in bladder cancer; (E) Common mutation sites in the 360 amino acids in PRR11; (F) Mutation frequencies of relevant genes in the PRR11‐LOW and PRR11‐HIGH groups. *p* < 0.05; **p < 0.01; ***p < 0.001; and ****p < 0.0001.

Through the cBioPortal database, we analyzed the mutational landscape of PRR11. The most common types of genetic alterations are amplification, mutation, and deep deletion (Figure 3A). Amplification of PRR11 was predominant in breast cancer, Pleural Mesothelioma, cholangiocarcinoma, and pheochromocytoma, while deep deletion was prevalent in prostate cancer. In bladder cancer, the main alteration of PRR11 was amplification (Amplification = 1.95%, Mutation = 0.73%). The primary type of mutation in PRR11 is a missense mutation, and the next is a code‐shift deletion. The most common types of mutations are single nucleotide polymorphisms (SNPs), and the mutation of cytosine (C) to thymine (T) is the most frequent single nucleotide mutation (Figure 3C,D). We identified a total of 360 amino acid sites, and the most common mutation site was E23Kfs*9 (Figure 3E). DNA repair is a complex mechanism that maintains genomic stability and integrity by detecting and eliminating abnormal chromosome sequences and structures. Tumor cells can evade some treatment strategies through mismatch repair (MMR) mechanisms, thus maintaining the self‐sustainability of tumor cells [25, 26]. A positive correlation was observed between PRR11 expression and the expression of MMR genes (Figure 3B).

In the TCGA‐BLCA dataset, the PRR11‐high group (*n* = 205) had a higher frequency of mutations in TP53 (60.8%), TTN (49.2%), and KMT2D (34.7%), whereas the low‐expression group (*n* = 204) showed a higher frequency of mutations in TTN (46.0%), TP53 (37.4%), and KDM6A (28.3%) (Figure S1D,E). The top mutated genes with the most significant differences were TP53, KMT2D, and RB1 in both groups (Figure 3F). According to the UALCAN database, PRR11 expression noticeably increases in patients with P53 mutations (Figure 3D). Overall, there is a significant association between PRR11 expression and genomic instability.

### 3.4 Enrichment Analysis of PRR11

**FIGURE 4:**
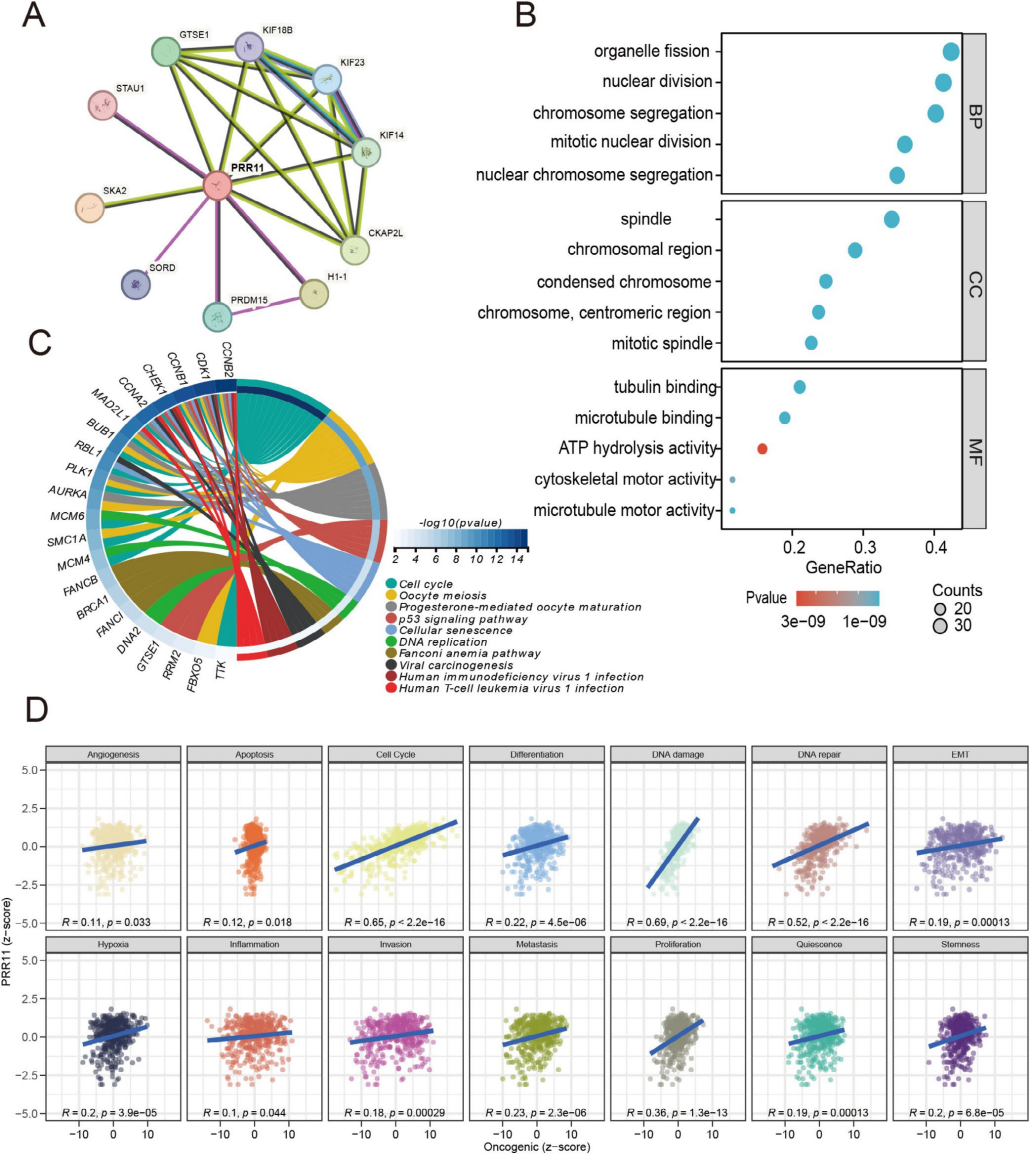
Correlation analysis and enrichment analysis of PRR11: (A) Protein–protein interaction analysis of PRR11 by the STRING database; (B) GO analysis of PRR11‐associated genes; (C) KEGG analysis of PRR11‐associated genes; (D) Correlation analysis of PRR11 with 14 functional states obtained from the CancerSEA database. *p* < 0.05; **p < 0.01; ***p < 0.001; and ****p < 0.0001.

To speculate on the function of PRR11, we performed a correlation analysis of PRR11 and an enrichment analysis. Based on the STRING database, we obtained 10 proteins closely associated with PRR11 (Figure 4A). Meanwhile, we retrieved the top 100 genes related to PRR11 from the GEPIA database and performed GO and KEGG analyses. GO analysis results showed that PRR11 was tightly associated with chromosome segregation, nuclear division, and organelle fission in biological processes (Figure 4B). KEGG analysis results suggested that pathways such as the Cell Cycle, Oocyte meiosis, and the p53 signaling pathway were strongly related to PRR11 (Figure 4C). By analyzing the CancerSEA database, we further observed that PRR11 was positively correlated with the cell cycle (Figure 4D, *R* = 0.65).

### 3.5 PRR11 Promotes the Proliferation, Metastasis, and Invasion of Bladder Cancer Cells

**FIGURE 5:**
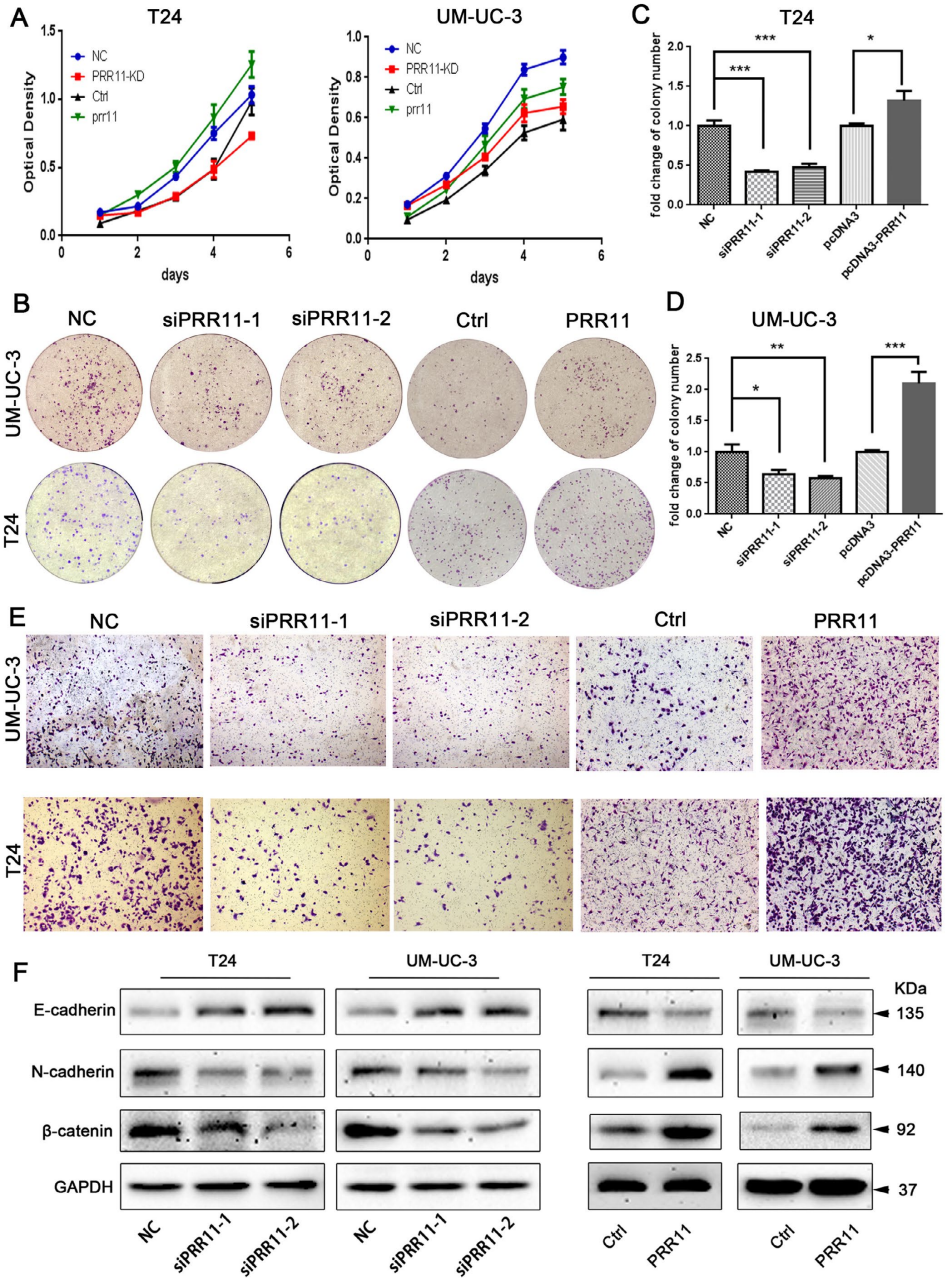
PRR11 promotes proliferation in bladder cancer cell lines: (A) MTT assay to analyze the proliferation ability of cells after PRR11‐specific siRNAs or overexpression of plasmid in both cell lines; NC and Ctrl were used as negative controls, respectively; (B–D) Reduced cell clonogenicity after the knockdown of PRR11 and increased cell cloning after the overexpression of PRR11 in T24 and UM‐UC‐3; (E) Transwell migration assay to verify cell migration and invasion after PRR11‐specific siRNAs or overexpression of the plasmid; NC and Ctrl were used as negative controls; (F) Changes in EMT pathway‐associated proteins after the knockdown or overexpression of PRR11. *p* < 0.05; **p < 0.01; ***p < 0.001; and ****p < 0.0001.

To explore the effect of PRR11 on bladder cancer, we constructed PRR11 silencing and overexpressing bladder cancer cell lines. The MTT assay showed that PRR11 knockdown (KD) inhibited the growth of T24 and UM‐UC‐3 cells, whereas PRR11 overexpression could promote proliferation (Figure 5A). Colony Formation Assay showed that in both cell lines, the knockdown of PRR11 resulted in a reduction of cell colonization, while the overexpression of PRR11 promoted colony formation (Figure 5B–D).

Metastasis is one of the characteristics of malignant tumors [27]. The Transwell migration assay demonstrated that PRR11 knockdown inhibited the migration and invasion of T24 and UM‐UC‐3 cells. Conversely, overexpression of PRR11 led to increased migration and invasion (Figure 5E). EMT is tightly associated with the metastasis of primary tumors [28, 29]. When PRR11 was knocked down, N‐cadherin and β‐catenin contents significantly decreased, and E‐cadherin content increased. Also, the opposite phenomenon was observed after overexpression of PRR11 (Figure 5F). We speculate that PRR11 may promote bladder cancer progression by affecting the related proteins in the EMT pathway.

**FIGURE 6:**
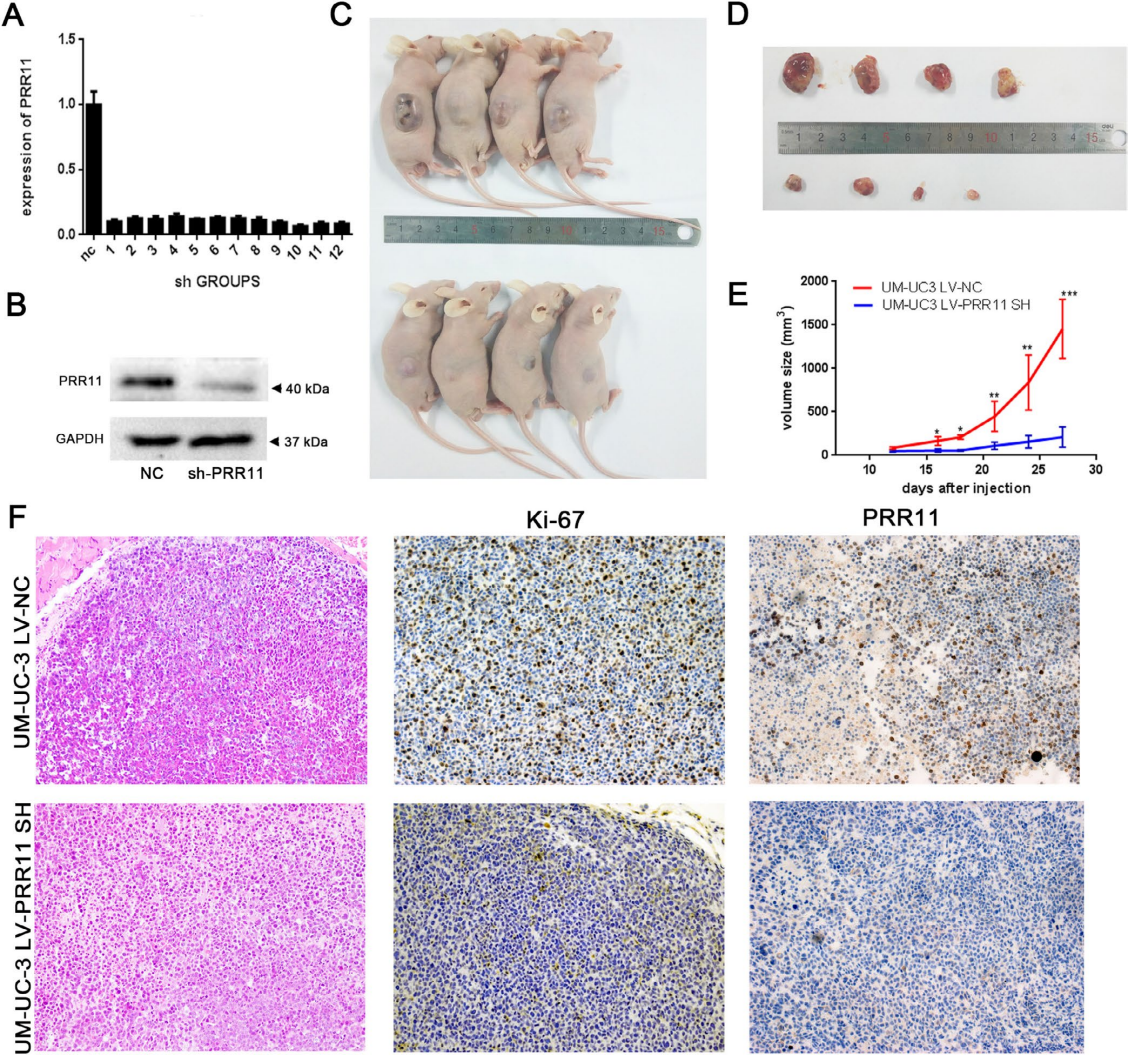
PRR11 accelerated the progression of BLCA in vivo: The knockdown effect of PRR11 in UM‐UC‐3 was verified by RT‐PCR (A) and WB experiments (B); (C, D) Naked eye observation of subcutaneous tumors; (E) Changes in tumor volume were recorded every 3 days in the PRR11 knockout and control groups; (F) HE and immunohistochemical staining of Ki‐67 and PRR11 in the NC and shPRR11 groups. *p* < 0.05; **p < 0.01; ***p < 0.001; and ****p < 0.0001.

To determine the role of PRR11 in vivo, we constructed a PRR11‐stabilized silenced UM‐UC‐3 cell line and verified the knockdown efficiency (Figure 6A); we also detected variations in the PRR11 protein levels (Figure 6B). An animal model was established by injecting the PRR11 stably silenced UM‐UC‐3 cell line into Balb/c nude mice. The tumor volume in the shPRR11 group was smaller than that in the control group, indicating that the depletion of PRR11 inhibited tumor growth (Figure 6C–E). Immunohistochemistry results indicated that the percentage of Ki‐67‐positive cells in the sh‐PRR11 group was lower (Figure 6F). In summary, PRR11 promotes BLCA progression in vitro and in vivo.

### 3.6 Silencing PRR11 Induces S‐Phase Arrest in BLCA

**FIGURE 7:**
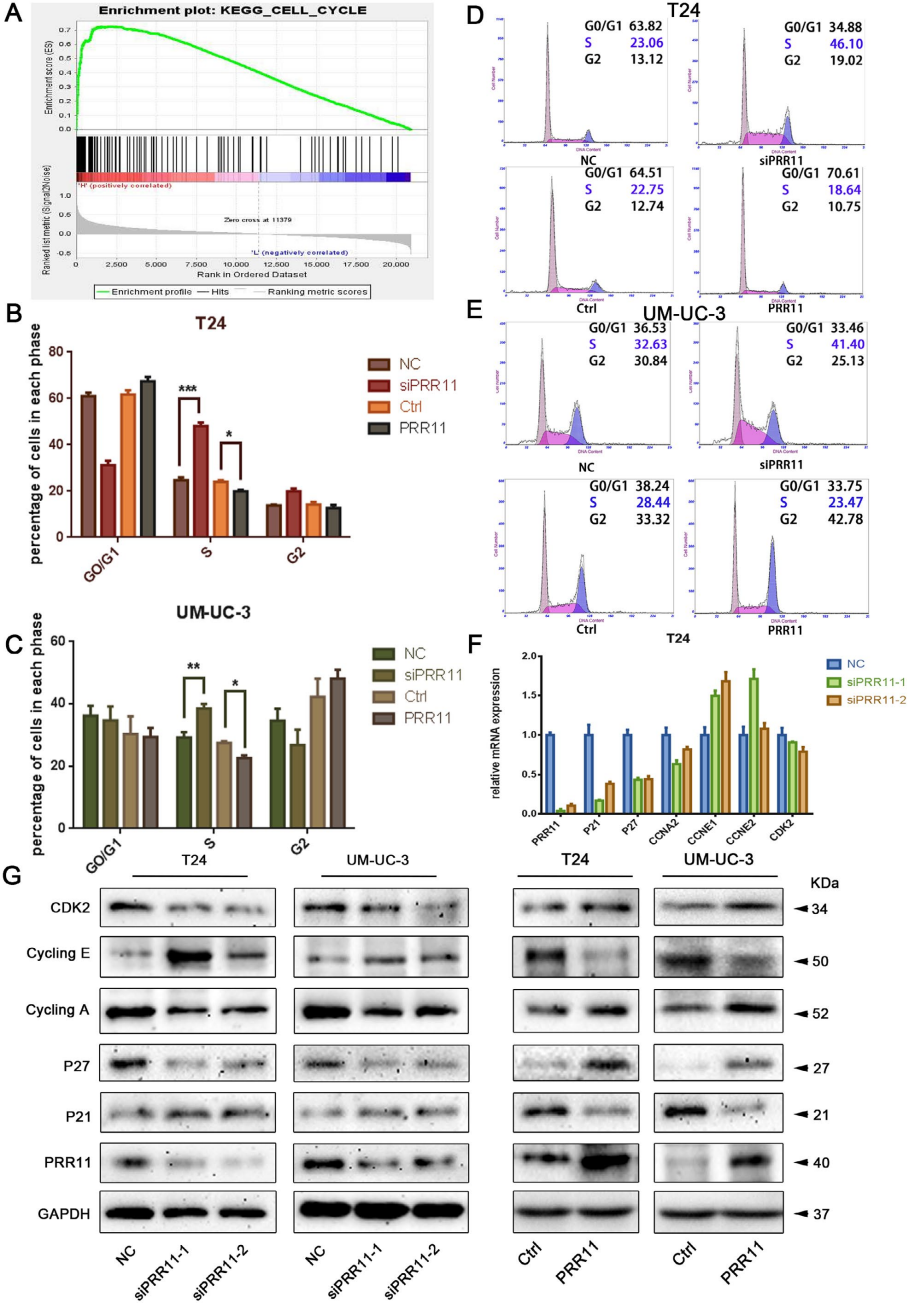
Silencing PRR11 induces S‐phase blockade in bladder cancer: (A) GSEA showing that PRR11 is associated with the cell cycle pathway; (B–E) Changes in cell cycle distribution of two bladder cancer cells after knockdown or overexpression of PRR11 detected by Flow Cytometry; (F) Changes in cell cycle‐regulated genes after knockdown or overexpression of PRR11 in T24 cell lines detected by RT‐PCR; (G) Variation of cell cycle‐associated regulatory proteins in two cell lines after knockdown or overexpression of PRR11. *p* < 0.05; **p < 0.01; ***p < 0.001; and ****p < 0.0001.

To explore the potential mechanism of PRR11, we performed gene set enrichment analysis (GSEA). PRR11 was enriched in the cell cycle pathway (Figure 7A and Figure S1F,G). Therefore, we postulated that PRR11 might promote tumor growth by affecting the cell cycle. Flow cytometry revealed that when PRR11 was knocked down, the distribution of the cell cycle phases changed significantly, with an increased proportion of cells in the S phase. The T24 cell line exhibited more obvious changes. In contrast, after PRR11 overexpression, the proportion of cells in the S phase decreased in both cell lines (Figure 7B–E).

Next, we verified the relationship between PRR11 and S‐phase‐related proteins. PCR and Western blot showed that CCNA and CCNE are essential regulatory proteins for the S phase and showed different trends (Figure 7F,G). When PRR11 was silenced, CCNE and p21 were upregulated, while CCNA, CDK2, and p27 were downregulated; the opposite phenomenon occurred when PRR11 was overexpressed. In summary, PRR11 affects bladder cancer progression by modulating the G1/S phase transition through the regulation of cell cycle‐related proteins.

## 4 Discussion

BLCA is a highly heterogeneous urologic malignancy, with incidence increasing annually worldwide [1]. Although standardized treatments, such as radical cystectomy and cisplatin‐based chemotherapy regimens, are essential for managing locally advanced and metastatic bladder cancer, their overall efficacy is limited, and the prognosis for patients resistant to chemotherapy remains poor [30]. Emerging strategies, including immunotherapy, targeted therapies, and cell cycle‐blocking treatments, offer new possibilities for treatment. Immune checkpoint inhibitors, such as PD‐1/PD‐L1 inhibitors, have shown success in treating metastatic bladder cancer [31]. In targeted therapy, FGFR3 inhibitors and VEGF inhibitors have also demonstrated efficacy [32, 33]. Novel combination therapies and precision medicine approaches, such as gene sequencing‐guided individualized treatment, are becoming increasingly important in bladder cancer management. These advancements provide hope for improving patient outcomes. Therefore, a comprehensive analysis of the pathological mechanisms and the identification of new therapeutic targets is essential for advancing treatment strategies.

Alterations of cell cycle regulatory mechanisms result in abnormal cell proliferation and genomic instability, ultimately leading to the malignant transformation of normal tissues. Targeting specific phases of the cell cycle is an effective strategy for inhibiting tumor growth by inducing apoptosis. CDK4/6 inhibitors inhibit tumor cell proliferation by blocking the G1/S phase transition, such as Ribociclib, Palbociclib, and Abemaciclib [34, 35, 36]. The S‐phase and G2/M phase are critical for DNA replication and cell division, and drugs like Gemcitabine and Docetaxel induce apoptosis by blocking these stages [37]. Gemcitabine, in particular, has proven effective as part of standard chemotherapy regimens [38]. In bladder cancer, identifying molecular markers closely related to the cell cycle process is of vital importance for early diagnosis and individualized treatment.

PRR11, a proline‐rich protein‐coding gene, is closely associated with the cell cycle [7]. The PRR11 protein consists of a nuclear localization signal (NLS), a zinc finger structural domain (ZFD), and two proline‐rich regions (PR) [8]. The potential therapeutic value of targeting PRR11 has been validated in a variety of cancers. Based on bioinformatics analysis and experimental validation, we have thoroughly explored the mechanism and therapeutic potential of PRR11 in bladder cancer.

By analyzing and processing patient expression data from the TCGA and GEO databases and validating them with RT‐PCR and Immunohistochemistry, we revealed that PRR11 was aberrantly overexpressed in tumor tissues. Notably, its expression increased in accordance with advancing clinical grade. Additionally, patients in the PRR11 high‐expression group exhibited lower survival rates, and COX regression analysis confirmed that PRR11 serves as an independent risk indicator for patient prognosis. These findings suggest that PRR11 could function as a specific biomarker and represent a potential target for clinical intervention in bladder cancer.

Gene mutations, or changes in the genetic material DNA, can lead to altered gene function. In cancer research, genetic mutations frequently involve tumor suppressor genes and oncogenes that affect cell growth, differentiation, and apoptosis [39]. The TP53 gene is an important tumor suppressor that maintains essential cellular functions and genomic stability [40]. As a G1/S checkpoint regulator, TP53 terminates the cell cycle and repairs DNA replication when DNA is damaged, and its mutation could promote tumorigenesis [41]. Our study found that the mutation rate of TP53 was as high as 60.08% in patients with high PRR11 expression. Moreover, we observed that TP53 mutations significantly promoted PRR11 expression. This interaction between TP53 and PRR11 in cancer could help us better understand the mechanisms of tumorigenesis and provide essential evidence for relevant therapeutic strategies.

In our study, silencing PRR11 suppressed the proliferation of bladder cancer cells, while overexpression of PRR11 enhanced their proliferative capacity, confirming the role of PRR11 in enhancing cell growth. Transwell migration assays further revealed that silencing PRR11 reduced invasion and migration. Metastasis is a crucial marker of cancer progression. EMT is closely associated with poor prognosis, characterized by the loss of epithelial cell properties and the acquisition of mesenchymal cell properties, which promotes biological aggressiveness [42, 43]. Overexpression of PRR11 in T24 cells promoted EMT, as evidenced by increased N‐Cadherin expression and decreased E‐Cadherin expression. Thus, PRR11 appears to drive both tumor growth and distant spread by promoting EMT in bladder cancer. In addition, we found a significant disparity in PRR11 expression among different pathology types, with higher PRR11 expression observed in recurrent patients. High PRR11 expression may increase the risk of transformation from non‐muscle‐invasive bladder cancer (NMIBC) to muscle‐invasive bladder cancer (MIBC), further driving the invasive and metastatic behavior of the tumor [44]. Bladder cancers of different tissue types may exhibit varying dependencies on the biological aggressiveness of EMT [45, 46]. For example, small cell carcinomas, which have higher invasiveness and metastatic potential, may rely more heavily on EMT‐related mechanisms, with PRR11 potentially playing an important role in this process. Further investigation into the molecular mechanisms of PRR11 in EMT regulation across different tissue types will facilitate the development of precision‐targeted therapies for specific subtypes.

Using the STRING database, we identified 10 proteins related to PRR11 and constructed a PPI network. This analysis revealed that PRR11 is closely associated with several key proteins, including KIF family proteins, STAU1, SKA2, and CKAP2L. KIF family proteins are essential for intracellular trafficking, cytokinesis, and mitosis. They maintain cellular function and structural stability by transporting organelles, vesicles, and protein complexes along microtubules. Studies have shown that KIF14 and KIF23 are aberrantly expressed in bladder cancer [47]. SKA2 is involved in the regulation of the cell cycle, especially during the G2/M transition. Its loss of function or aberrant expression may lead to chromosome segregation errors, contributing to tumorigenesis and progression [48]. CKAP2L plays a critical function in the reorganization of the cytoskeleton. The stability of the cytoskeleton is essential for maintaining cell morphology and regulating migratory ability. Aberrant expression of CKAP2L may lead to the reorganization of the cytoskeleton, thereby enhancing the invasion ability of cancer [49]. This network provides insights into the molecular interactions involving PRR11, suggesting that these associated proteins may also play significant roles in the progression of bladder cancer.

To understand the role of PRR11 in tumor growth, we performed functional enrichment analysis of related genes in the GEPIA database. GO analysis revealed that PRR11 is closely associated with biological processes such as chromosome segregation and organelle fission, which are important aspects of mitosis. Furthermore, KEGG analysis showed that PRR11 and its related genes were enriched in pathways such as cell cycle and oocyte meiosis. In addition, we assessed the association of PRR11 with 14 cellular phenotypes through the analysis of CancerSEA. The results indicated that PRR11 plays important roles in functional states such as cell cycle, DNA damage, and DNA repair. To further elucidate the molecular mechanisms through which PRR11 influences bladder cancer, we performed GSEA, which confirmed that the cell cycle is the primary pathway through which PRR11 drives cancer progression. These findings reveal the potential mechanisms of PRR11 and provide new perspectives for future research and therapeutic strategies.

Cell cycle arrest is one of the emerging therapeutic strategies for tumor treatment [50]. DNA synthesis and mitosis play vital functions in maintaining the malignant proliferation of tumor cells [51]. Some antitumor drugs, such as Gemcitabine and Docetaxel, can achieve antitumor effects by arresting tumor cells in S‐phase and inducing them to initiate apoptosis [38, 52]. Our results indicate that the knockdown of PRR11 induces S‐phase arrest. When S‐phase is blocked, some relevant signaling pathways are activated, provoking the cell death‐related cascade response and inhibiting tumor cell proliferation. Western blot analysis showed that silencing PRR11 could promote the expression of CCNE and decrease CDK2 and CCNA. The complex formed by CDK2 and CCNE promotes the G1/S transition. In contrast, CCNE is rapidly degraded after entering the S phase, and CCNA functions in the internal processes of S phase [53, 54, 55]. Depletion of PRR11 causes a deficiency of CCNA proteins, leading to cell cycle disruption in S phase and exerting tumor suppressor effects. Thus, we predict that targeting PRR11 could effectively reduce the number of tumor cells by blocking S‐phase progression, demonstrating its profound potency in tumor therapy.

Although there are interesting findings, this study still has some limitations:(1) More clinical data are needed to validate the prognostic impact of PRR11 in bladder cancer; (2) There is also a need to explore whether PRR11 promotes bladder cancer progression through other mechanisms; (3) Potential drugs targeting PRR11 in bladder cancer must be investigated.

## 5 Conclusion

Our study confirmed that PRR11 is overexpressed in bladder cancer tissues, with its expression levels increasing as the tumor progresses. We also discovered that PRR11 promotes malignant progression by driving epithelial –mesenchymal transition and facilitating the G1/S cell cycle transition. These findings enhance our understanding of PRR11 and offer new molecular insights for developing targeted cell cycle therapies.

## Author Contributions

**Lu Wang:** data curation (lead), formal analysis (lead), investigation (lead), methodology (lead). **Zengshun Kou:** data curation (equal), formal analysis (equal), methodology (equal), writing – original draft (equal). **Jiaxi Zhu:** resources (supporting), software (supporting), validation (supporting), writing – review and editing (supporting). **Xiu Zhu:** conceptualization (supporting), resources (supporting), visualization (supporting), writing – review and editing (supporting). **Lei Gao:** project administration (supporting), software (supporting). **Hai Zhu:** conceptualization (lead), funding acquisition (lead), supervision (lead), writing – review and editing (lead).

## Ethics Statement

This study was approved by the Ethical Review Committee of Zhongnan Hospital of Wuhan University with all patients informed consent signed (approval number: 2015029).

## Conflicts of Interest

The authors declare no conflicts of interest.

## Supporting information


Data S1.

Data S2.

Data S3.

## Data Availability

All datasets used in this study are available upon reasonable request from the corresponding author.
