# Supplementary material for: PRR11 Promotes Bladder Cancer Growth and Metastasis by Facilitating G1/S Progression and Epithelial‐Mesenchymal Transition
Source: Cancer Med. 2025 Mar 10;14(5):e70749. doi: 10.1002/cam4.70749 (PMC11891932; doi:10.1002/cam4.70749)
Supplement: Supplementary file 1 — Data S1. [file CAM4-14-e70749-s002.docx]

**Additional relevant information**

**TABLE 1.Clinical information for patients in TCGA**

| Characteristics | Low expression of PRR11 | High expression of PRR11 | P value |
| --- | --- | --- | --- |
| n | 206 | 206 |  |
| Pathologic T stage, n (%) |  |  | 0.634 |
| T1&T2 | 65 (17.2%) | 58 (15.3%) |  |
| T3 | 95 (25.1%) | 101 (26.7%) |  |
| T4 | 32 (8.5%) | 27 (7.1%) |  |
| Pathologic N stage, n (%) |  |  | 0.531 |
| N0 | 116 (31.5%) | 122 (33.2%) |  |
| N1 | 20 (5.4%) | 26 (7.1%) |  |
| N2&N3 | 45 (12.2%) | 39 (10.6%) |  |
| Pathologic M stage, n (%) |  |  | 0.747 |
| M0 | 111 (52.4%) | 90 (42.5%) |  |
| M1 | 5 (2.4%) | 6 (2.8%) |  |
| Pathologic stage, n (%) |  |  | 0.607 |
| Stage I | 3 (0.7%) | 1 (0.2%) |  |
| Stage II | 68 (16.6%) | 61 (14.9%) |  |
| Stage III | 67 (16.3%) | 75 (18.3%) |  |
| Stage IV | 67 (16.3%) | 68 (16.6%) |  |
| Gender, n (%) |  |  | 0.502 |
| Female | 57 (13.8%) | 51 (12.4%) |  |
| Male | 149 (36.2%) | 155 (37.6%) |  |
| Age, n (%) |  |  | 0.321 |
| <= 70 | 111 (26.9%) | 121 (29.4%) |  |
| > 70 | 95 (23.1%) | 85 (20.6%) |  |
| BMI, n (%) |  |  | 0.092 |
| <= 25 | 86 (23.8%) | 66 (18.2%) |  |
| > 25 | 100 (27.6%) | 110 (30.4%) |  |
| Subtype, n (%) |  |  | 0.004 |
| Non-Papillary | 123 (30.2%) | 150 (36.9%) |  |
| Papillary | 81 (19.9%) | 53 (13%) |  |
| Smoker, n (%) |  |  | 0.001 |
| No | 70 (17.5%) | 39 (9.8%) |  |
| Yes | 133 (33.3%) | 157 (39.3%) |  |

**TABLE 2.Clinical information for patients with bladder cancer**

| **Patients** | **Genders** | **Age** | **Clinical Stages** | **Pathological classification** |
| --- | --- | --- | --- | --- |
| **1** | Female | 63 | T1 | 3 |
| **2** | Male | 76 | T1 | **2** |
| **3** | Male | 52 | T1 | **2** |
| **4** | Female | 68 | T1 | **2** |
| **5** | Male | 65 | T1 | **2** |
| **6** | Male | 73 | T1 | **2** |
| **7** | Male | 61 | T1 | **3** |
| **8** | Male | 48 | T2 | **2** |
| **9** | Male | 63 | T2 | **3** |
| **10** | Male | 51 | T2 | **3** |
| **11** | Female | 87 | T2 | **2** |
| **12** | Male | 69 | T2 | **3** |
| **13** | Male | 61 | T2 | **3** |
| **14** | Male | 48 | T3 | **2** |
| **15** | Male | 73 | T3 | **3** |
| **16** | Male | 64 | T3 | **3** |
| **17** | Female | 68 | T3N1 | **3** |
| **18** | Male | 59 | T3N1 | **3** |
| **19** | Male | 74 | T4 | **3** |

**TABLE 3.Sequence of PCR upstream and downstream primers**

| Gene | Forward primer 5'-3' | Reverse primer5'-3' |
| --- | --- | --- |
| GAPDH | TGCACCACCAACTGCTTAG | GATGCAGGGATGATGTTC |
| PRR11 | GAAGCTGGCTAACATCATCCTG | CTCTGGGTTATGCAGTTCTGG |
| CDK2 | CCAGGAGTTACTTCTATGCCTGA | TTCATCCAGGGGAGGTACAAC |
| CCNA2 | CGCTGGCGGTACTGAAGTC | GAGGAACGGTGACATGCTCAT |
| P21 | CGATGGAACTTCGACTTTGTCA | GCACAAGGGTACAAGACAGTG |
| P27 | TAATTGGGGCTCCGGCTAACT | TGCAGGTCGCTTCCTTATTCC |
| CCNE1 | GCCAGCCTTGGGACAATAATG | CTTGCACGTTGAGTTTGGGT |
| CCNE2 | TCAAGACGAAGTAGCCGTTTAC | TGACATCCTGGGTAGTTTTCCTC |

**TABLE 4.Information about the primary antibodies**

| **Antibody** | **Species** | **Molecular weight(Kda)** | **Dilution ratio** | **Provider** |
| --- | --- | --- | --- | --- |
| GAPDH | Mouse | 37 | 1:2000 | SANTA CRUZ, USA |
| PRR11 | Rabbit | 40 | 1:1000 | abclonal，China |
| PRR11 | Rabbit | 40 | 1:1000 | abclonal，China |
| cyclinA1+A2 | Rabbit | 49,52 | 1:1000 | abcam，USA |
| cyclinE1 | Rabbit | 50 | 1:1000 | abcam，USA |
| P21 | Rabbit | 21 | 1:1000 | abcam，USA |
| P27 | Rabbit | 27 | 1:1000 | abcam，USA |
| CDK2 | Rabbit | 34 | 1:1000 | abcam，USA |
| E-cadherin | Rabbit | 135 | 1:500 | CST，USA |
| N-cadherin | Rabbit | 140 | 1:500 | CST，USA |
| β-catenin | Rabbit | 92 | 1:1000 | CST，USA |

**TABLE 5. Primers for gene knockdown**

| GENE | sense（5'-3'） |
| --- | --- |
| shPRR11-1 | GCACGGAAUCCACUAGUUAtt |
| shPRR11-2 | GGCCUUAAGGAGAAAGUUUtt |
| shPRR11-3 | CCCAGAGUUUAGAAGUAUUtt |

**Information about secondary antibodies：**

Goat anti-Rabbit IgG（H+L） SUNGENE BIOTECH China

Goat anti-mouse IgG（H+L） SUNGENE BIOTECH China
