# Supplementary material for: PRR11 Promotes Bladder Cancer Growth and Metastasis by Facilitating G1/S Progression and Epithelial‐Mesenchymal Transition
Source: Cancer Med. 2025 Mar 10;14(5):e70749. doi: 10.1002/cam4.70749 (PMC11891932; doi:10.1002/cam4.70749)
Supplement: Supplementary file 2 — Data S2. [file CAM4-14-e70749-s001.docx]

**Supplementary figure**


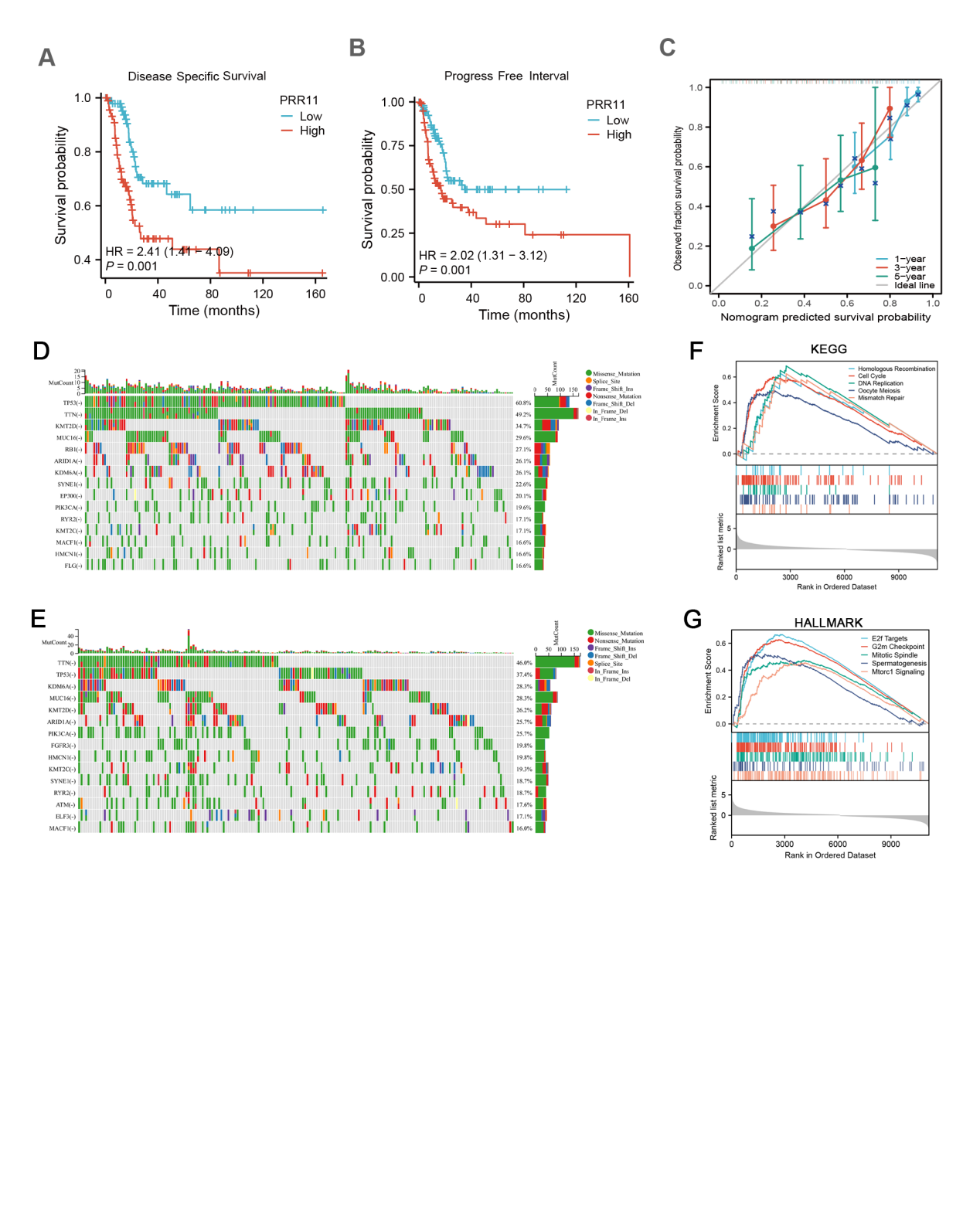


**Figure. Supplementary：**(A) K-M analysis of DSS for PRR11-LOW and PRR11-HIGH in BLCA; (B) K-M analysis of PFI for PRR11-LOW and PRR11-HIGH in BLCA; (C) Calibration curves for prognostic Nomogram; (D) Mutation of genes in PRR11 high expression group; (E) Mutations of genes in PRR11 low expression group; (F) Signaling pathways enriched by PRR11 through KEGG dataset in bladder cancer; (G) Signaling pathways enriched by PRR11 through HALLMARK dataset in bladder cancer.
